# P-1662. Antifungal Prophylaxis in Liver Transplants: Navigating the Optimal Duration

**DOI:** 10.1093/ofid/ofae631.1828

**Published:** 2025-01-29

**Authors:** Fabian Dammann, Shweta Anjan, Ana Vega, Yoichiro Natori, Gennaro Selvaggi, Rodrigo Vianna, Lilian M Abbo, Christine A Vu

**Affiliations:** Chase Brexton Health Care, Baltimore, Maryland; University of Miami Miller School of Medicine and Miami Transplant Institute, Jackson Health System, Miami, Florida; Jackson Memorial Hospital, Miami, Florida; University of Miami, Miami Transplant Institute, Jackson Health System, Miami, Florida; Miami Transplant Institute, Jackson Health System, Miami, Florida; Jackson Memorial Hospital/ Miami Transplant Institute, Miami, Florida; University of Miami Miller School of Medicine, Jackson Health System, Aventura, FL; Jackson Memorial Hospital, Miami, Florida

## Abstract

**Background:**

Invasive candidiasis (IC) is associated with a high mortality rate following liver transplantation (LT). Data on ideal duration of antifungal prophylaxis post-LT is limited. We investigated the effect of decreasing antifungal prophylaxis duration on the incidence of IC in LT recipients.

**Figure:**
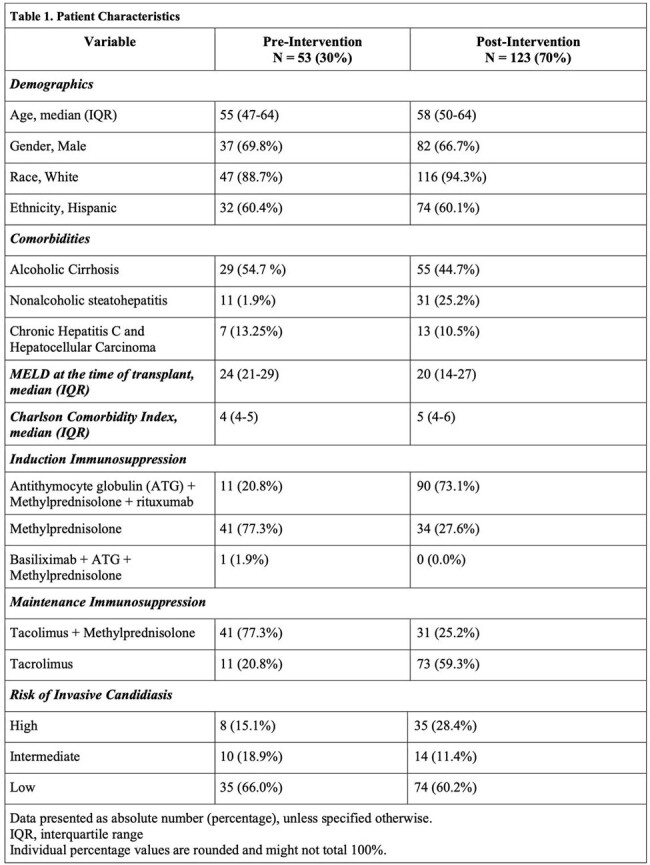

**Methods:**

Single-center, retrospective study was performed comparing two prophylaxis strategies in adult LT recipients at a large transplant center. In 2021, a stewardship intervention was made to decrease the duration of antifungal prophylaxis. In pre-intervention group (Jan 1 - Dec 31, 2020), patients received no prophylaxis (low risk), fluconazole for 14 days (intermediate risk), or micafungin for 14 days (high risk). For the post-intervention group (Jan 1 to Dec 31, 2022), this was decreased to fluconazole for 5 days (intermediate risk) or micafungin for 7 days (high risk). Primary outcome was incidence of IC, defined as isolation of *Candida spp.* from a sterile site. For risk factor assessment, Chi-square and Mann-Whitney U test were conducted as univariate analysis. Multivariate logistic regression model was developed based on univariate analysis. Log-rank test was utilized to compare the incidence between pre- and post- intervention era.

**Figure:**
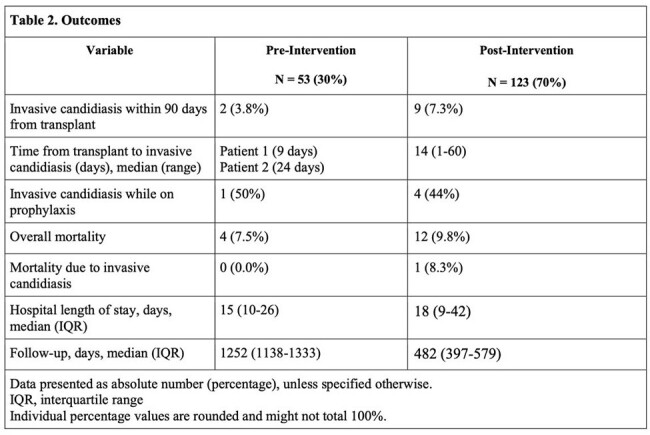

**Results:**

Of the 176 LT recipients included, 53 (30%) in the pre-intervention group and 123 (70%) in the post-intervention group (Table 1), 2 (3.8%) versus 9 (7.3%) patients developed IC respectively (p=0.37). Five (45.5%) patients developed IC while on prophylaxis. Overall mortality was 16 (9.0%), with one death related to IC (Table 2). In a univariate analysis, prolonged mechanical ventilation (p=0.002), re-opening of abdomen (p=0.023), ICU stay prior to transplant (p=0.036), prior antifungals (p=0.021), prior antibiotics (p=0.016), MELD > 30 (p=0.004) were associated with increased risk for IC in both groups. After a multivariate analysis, MELD > 30 (p=0.039, OR 3.94, 95%CI 1.07-14.49) and prolonged mechanical ventilation (p=0.020, OR 4.84, 95%CI 1.29-18.18) was associated with increased risk for IC.

**Conclusion:**

Decreasing the duration of antifungal prophylaxis in LT recipients did not significantly impact the incidence of IC. Further research is needed to refine antifungal stewardship strategies in this high risk patient population.

**Disclosures:**

**Yoichiro Natori, MD**, Eurofin/Viracor: Honoraria|Nobel Pharma: Advisor/Consultant

